# Personalized 3D-Printed Hybrid PDMS and PEEK Implants for Revisional Orbitomaxillary Reconstruction: A Translational Case-Based Technical Note

**DOI:** 10.3390/jfb17040197

**Published:** 2026-04-18

**Authors:** Goran Marić, Darko Solter, Blanka Doko Mandić, Jelena Škunca Herman, Zoran Vatavuk, Damir Godec, Davor Vagić, Alan Pegan

**Affiliations:** 1 Department of Ophthalmology, Univeristy Hospital Center Sestre Milosrdnice, Vinogradska Cesta 29, 10000 Zagreb, Croatia; 2 Department of Otorhinolaryngology and Head and Neck Surgery, University Hospital Center Sestre Milosrdnice, Vinogradska Cesta 29, 10000 Zagreb, Croatia; 3 Faculty of Mechanical Engineering and Naval Architecture, University of Zagreb, 10000 Zagreb, Croatia; damir.godec@fsb.unizg.hr

**Keywords:** PDMS, PEEK, personalized implant, 3D printing, revision maxillofacial surgery, revisional craniofacial reconstruction, translational biocompatible material, hybrid biomaterials, graded stiffness, diplopia

## Abstract

The reconstruction of complex orbitomaxillary defects requires biomaterials that can simultaneously provide structural stability, biocompatibility, and accurate restoration of facial volume and contour. While rigid polymers such as polyetheretherketone (PEEK) offer reliable mechanical support, they do not adequately replicate the viscoelastic behavior of soft tissues. This report presents a translational revision case employing a personalized hybrid biomaterial approach that combines a 3D-printed PEEK implant for structural orbital floor support with a patient-specific polydimethylsiloxane (PDMS) implant for malar volumetric augmentation. Reconstruction was planned using CT segmentation and contralateral mirroring. Patient-specific implants were subsequently designed using CAD/CAM techniques, combining a rigid PEEK implant for structural orbital support with a flexible PDMS implant for malar volumetric augmentation with complementary mechanical properties. Revision surgery included the removal of inadequately positioned titanium hardware, the release of incarcerated extraocular muscles, and the restoration of orbital anatomy and facial symmetry. Postoperative imaging demonstrated stable implant positioning and sustained orbitomaxillary stability. Despite successful anatomical reconstruction, residual functional sequelae, including strabismus related to the severity of the initial orbital trauma, persisted and were addressed separately in a staged manner, resulting in satisfactory ocular alignment and resolution of diplopia in primary gaze. This case underscores the complementary functional roles of rigid and elastic polymers and highlights the translational potential of PDMS as a permanent, patient-specific implant material for volumetric and contour restoration in craniofacial reconstruction.

## 1. Introduction

The reconstruction of complex orbitomaxillary defects remains one of the greatest challenges in cranio-maxillofacial surgery. Trauma, oncologic resections, and previous surgical interventions can lead to the extensive loss of hard and soft tissue support, often resulting in diplopia, ocular motility disturbances, pain, and significant facial asymmetry. Secondary or revision surgeries are particularly demanding due to scarred tissues, altered anatomy, and the presence of foreign materials, most commonly titanium plates and meshes [1,2].

Although titanium and polyetheretherketone (PEEK) implants have become standard materials in reconstructive maxillofacial surgery, their limitations are well recognized [3]. Titanium provides mechanical stability but lacks flexibility and may cause cold conduction, palpability, and contour irregularities [4]. PEEK, while more aesthetically favorable and radiolucent, remains rigid and does not fully replicate the viscoelastic nature of soft tissues [5]. This mismatch between rigid implants and compliant soft tissues represents a key limitation in achieving natural contour restoration. Consequently, there is a growing need for biomaterials that combine mechanical strength with elasticity, biocompatibility, and long-term stability. Medical-grade polydimethylsiloxane (PDMS) has been extensively used in other surgical disciplines due to its proven biocompatibility, flexibility, and chemical stability [6]. However, its application as a permanent implant material in craniofacial reconstruction remains limited, particularly in addressing soft-tissue contour deficiencies following skeletal reconstruction. The advent of computer-aided design and additive manufacturing (3D printing) now allows for the precise fabrication of personalized implants adapted to patient-specific anatomy [6,7]. Combining rigid PEEK for structural support with flexible PDMS for contour restoration represents a hybrid reconstructive strategy that better approximates the mechanical behavior of native tissues. This approach aims to bridge the gap between skeletal reconstruction and soft-tissue conformity.

Here, we present a complex revision case in which chronic diplopia, orbital pain, and facial asymmetry after inadequate primary fixation were successfully treated using personalized 3D-printed PEEK and PDMS implants in combination with autologous micro-fragmented fat grafting. To our knowledge, reports describing patient-specific hybrid PDMS–PEEK strategies in revision orbitomaxillary reconstruction remain scarce.

## 2. Case Presentation

### 2.1. Clinical History and Indication

A 27-year-old male patient was referred to our department with persistent oblique diplopia, painful eye movements and noticeable facial asymmetry after two orbital and midfacial reconstruction surgeries performed at an external institution.

In June 2022, the patient sustained severe polytrauma in a traffic accident, resulting in multiple comminuted neurocranial fractures, predominantly involving the right frontal and temporal bones, with complex viscerocranial fractures, including the maxilla, orbital floor, and an impression fracture of the right zygomatic bone. The injury was accompanied by subarachnoid hemorrhage, and the patient remained comatose for approximately three weeks.

Following stabilization of his neurological condition and resolution of intracranial bleeding, a primary orbitomaxillary reconstruction was performed at another institution. In the first procedure, a repositioning of the osteosynthesized zygomatic arch was performed and due to significant loss of orbital fat volume, multiple titanium strips and meshes were additionally inserted in an attempt to compensate for volume loss and to support the orbital floor. During the second reconstructive procedure, osteosynthesis and repositioning of the roof of the orbit, frontal bone, was performed. Although the early postoperative course was uneventful, within several weeks the patient developed persistent symptoms.

In August 2025, three years after the initial procedures, the patient presented to our UHC due to residual symptoms and signs related to the right eye and orbit—diplopia, orbital pain and unacceptable esthetic outcome.

Persistent oblique and vertical diplopia were present at all gaze positions. Clinical examination revealed restricted ocular motility, particularly in vertical (upward and downward) gaze, suggesting a combined neuromechanical disturbance related to prior trauma and reconstruction (consistent with partial oculomotor nerve paresis and possible entrapment of the inferior rectus muscle). These findings were corroborated by computed tomography (CT) of orbits and paranasal sinuses (PNS), which demonstrated right eyeball proptosis, irregular contouring of the malar region, inferior displacement of the orbital floor, and aberrant position of inferior rectus muscle due to mechanical incarceration between multiple titanium plates, one of which is placed above the muscle and reaches the very attachment point to the globe.

Daily right-sided orbital pain, which worsens with eye movement, was 7/10 on the visual analog scale (VAS) [8]. The unsatisfactory esthetic outcome was characterized by ocular misalignment, periorbital asymmetry, and flattening of the malar prominence on the right side.

Given the persistence of diplopia, chronic pain, and esthetic deformity, a revision orbitomaxillary reconstruction was indicated to relieve pain, restore orbital volume and ocular function and re-establish facial symmetry using personalized 3D-printed biocompatible implants.

### 2.2. Preoperative Planning

Comprehensive preoperative planning was undertaken to assess the extent of orbital deformity and to design patient-specific implants aimed at restoring anatomical structure and functional integrity. High-resolution computed tomography (CT) of the craniofacial region was performed in axial, coronal, and sagittal planes, followed by three-dimensional reconstruction of the orbitomaxillary complex.

**Figure 1:**
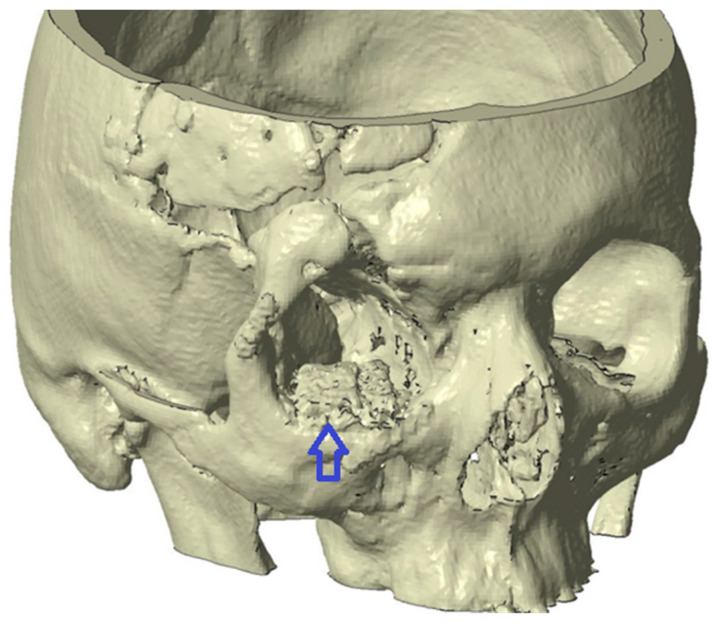
Three-dimensional reconstruction of the orbitomaxillary complex showing multiple overlapping titanium meshes (blue arrow) and secondary volume asymmetry compared with the contralateral side. Multiplanar CT analysis confirmed inferior displacement of the orbital contents and malar volume deficiency.

The analysis confirmed a residual deformity of the right orbital floor and malar region, including inferior displacement of the orbital contents, orbital fat atrophy, and secondary volume asymmetry compared with the contralateral side. Multiple overlapping titanium meshes from previous interventions were also identified (Figure 1). 

For virtual reconstruction, the unaffected (left) side was segmented and mirrored to serve as a geometric reference for the affected side. This approach enabled the generation of a patient-specific three-dimensional model that guided the restoration of orbital floor anatomy and malar projection, with the aim of achieving both functional orbital support and facial symmetry.

The finalized anatomical reconstruction was exported as stereolithographic (STL) files and used as the basis for subsequent implant design and fabrication [9,10,11,12,13,14,15].

### 2.3. Implant Design and Fabrication

Based on the preoperative analysis and virtual reconstruction, a hybrid reconstructive strategy was adopted using two distinct medical-grade biomaterials with complementary mechanical and biological properties: polyetheretherketone (PEEK) and polydimethylsiloxane (PDMS).

A rigid PEEK implant (VESTAKEEP i4 3DF) was designed for reconstruction of the orbital floor, providing high mechanical strength, dimensional stability, and long-term structural support. Its radiolucency enables artifact-free postoperative imaging, while its established biocompatibility and chemical inertness make it suitable for permanent implantation in load-bearing craniofacial regions [4].

In parallel, a flexible PDMS implant (NuSil MED-4244) was developed for augmentation of the malar region and restoration of facial contour. Due to its viscoelastic properties, PDMS allows close adaptation to the underlying bone while providing a smooth transition to the overlying soft tissues. This property was considered particularly advantageous in addressing contour deformities and soft-tissue deficiencies that persist despite adequate skeletal reconstruction.

During digital implant design, a volumetric negative offset (undercorrection) of approximately 20% was intentionally applied to the PDMS component to account for expected postoperative soft-tissue adaptation and fibrous capsule formation. This adjustment was based on surgical experience and aimed to achieve the desired final contour following biological integration.

Implant geometries were generated using InVesalius 3.1 (CTI Renato Archer, Campinas, SP, Brazil) and further refined in Rhinoceros 7 (Robert McNeel & Associates, Seattle, WA, USA) to ensure precise adaptation to the native anatomy. The orbital implant was designed as a patient-specific replica of the contralateral orbital floor, with thickness ranging from 1.5 to 2.0 mm, while preserving sufficient rigidity for load-bearing support. All digital steps were controlled by the surgical team to ensure direct integration between planning and intraoperative execution.

**Figure 2:**
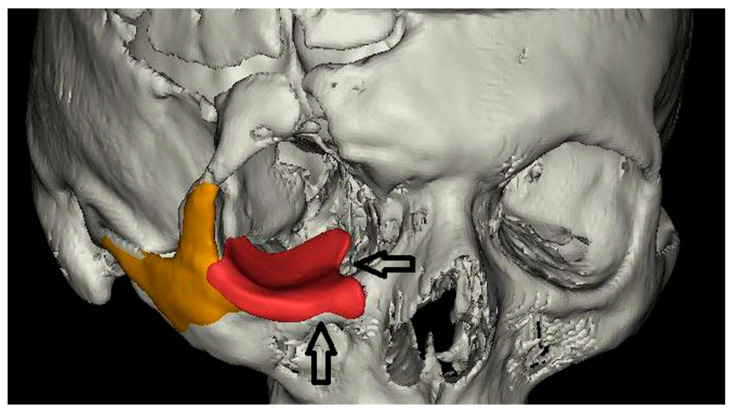
PEEK implant (red) for reconstruction of the orbital floor and a flexible PDMS implant (orange) for restoration of malar projection and facial symmetry. Rounded relief zones incorporated into the PEEK implant to avoid the infraorbital foramen and nerve (down arrow) and the origin of the inferior oblique muscle (right arrow).

Special attention was given to preserving the origin of the inferior oblique muscle and avoiding compression of the infraorbital foramen and nerve, which was achieved by incorporating rounded relief zones within the implant geometry (Figure 2).

Both implants were exported as STL models and fabricated using additive manufacturing technologies. The PEEK implant was produced via high-temperature fused filament fabrication (FFF) using parameters appropriate for implant-grade materials, ensuring optimal interlayer adhesion and dimensional stability in accordance with ASTM F2026 standards.

The PDMS implant was fabricated by casting into a custom negative mold derived from the digital model, followed by thermal curing at 140 °C for 1 h to achieve complete cross-linking of the elastomer.

The silicone material complies with ISO 10993 standards for biocompatibility of implantable devices. After surface finishing and cleaning, both implants were sterilized using steam autoclaving at 121 °C and 15 psi for 30 min using a validated institutional protocol, without observable dimensional deformation or surface degradation [16,17,18,19].

The final constructs underwent dimensional verification and sterile packaging prior to surgery to ensure precise intraoperative fit and immediate clinical applicability.

### 2.4. Surgical Procedure

The revision orbitomaxillary reconstruction was performed under general endotracheal anesthesia following standard aseptic preparation and ophthalmic protection. A subciliary incision was used to access the orbital rim and floor, followed by meticulous subperiosteal dissection of the lower eyelid and malar tissues. The surgical field was extended inferiorly to expose the orbital floor (roof of the maxillary sinus) and the previously placed titanium meshes.

**Figure 3:**
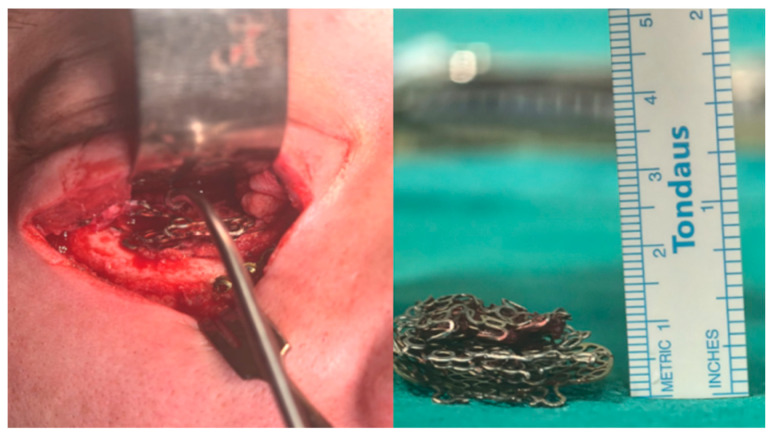
Surgical procedure. Multiple titanium plates in situ and after removal.

A total of five titanium plates of varying sizes were identified and meticulously removed (Figure 3) under magnification using surgical loupes. Fibrotic adhesions were dissected sharply, and the orbital contents were mobilized with extreme care. The inferior rectus and inferior oblique muscles were disincarcerated and released from the surrounding scar tissue, to ensure preservation of their integrity as well as that of the optic nerve, oculomotor nerve, and other vital orbital structures.

Following complete decompression of the orbital contents, the orbital floor defect (roof of the maxillary sinus) was reconstructed using the custom-made PEEK implant. The implant was precisely fitted to the anatomic contour and secured with two titanium screws placed along the inferior orbital rim, achieving stable fixation and restoration of orbital volume.

**Figure 4:**
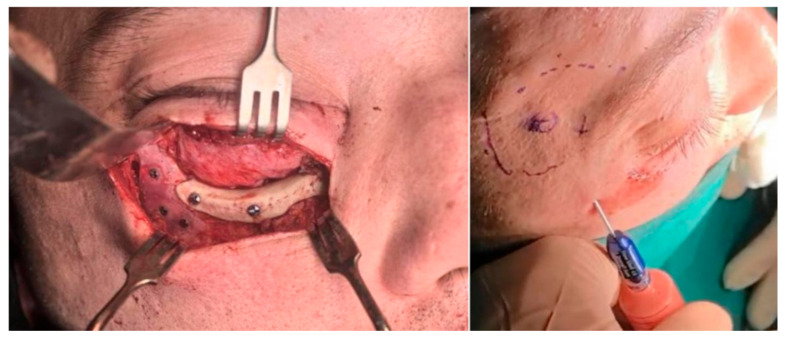
(**Left**) Intraoperative view of PDMS and PEEK implants fixed with titanium screws. (**Right**) Micro-fragmented fat grafting to the orbital cavity for volumetric restoration in the periocular temporal region to address contour irregularities (outlined).

The custom-made PDMS implant was then positioned in the malar region, corresponding to the area of post-traumatic depression caused by the previous impression fracture of the zygomatic bone. Its geometry allowed smooth contour restoration and soft-tissue adaptation without the need for osteotomy or bone repositioning. The implant was secured with three titanium screws to prevent migration and ensure positional stability. After implant placement, an autologous fat graft was harvested from the periumbilical region using low-pressure liposuction. The aspirated fat was micro-fragmented and injected into the orbital region (6 mL), to address orbital fat atrophy and optimize volume restoration. An additional 12 mL of processed fat was injected subcutaneously into the frontal and temporal regions to correct contour irregularities and improve soft-tissue symmetry (Figure 4).

Hemostasis was achieved, and the surgical field was irrigated with sterile saline. The incision was closed in anatomical layers using resorbable sutures for the deeper planes and a non-resorbable skin suture, which was removed on postoperative day seven. The procedure was completed without intraoperative complications.

## 3. Results (Postoperative Follow-Up Course and Outcomes)

### 3.1. Early Postoperative Course

The immediate postoperative course was uneventful, with no evidence of bleeding, infection, or wound dehiscence. The patient was discharged on postoperative day four with satisfactory wound healing and no early complications. At the one-month follow-up the incision had completely healed, and the early postoperative mild periorbital edema completely resolved spontaneously. No signs of implant migration, inflammatory reaction, or sensory deficit were observed.

### 3.2. Residual Strabismus and Secondary Ophthalmological Management

Preoperative ophthalmological assessment revealed a complex ocular misalignment with persistent diplopia and right eye exohypotropia. The vertical deviation measured 30 prism diopters (PD) in primary gaze, with an associated horizontal exodeviation of 30–35 PD. Ocular motility was markedly restricted, particularly in vertical gaze (restricted elevation) and binocular function was absent. A mild upper eyelid ptosis of the right eye was also present. These findings were consistent with a combined post-traumatic neuro-mechanical ocular motility disorder related to the severity of the initial orbital injury and previous reconstructive procedures. 

At the beginning of the revision orbitomaxillary surgery forced duction testing suggested limited passive globe mobility, compatible with an adaptive restrictive component. Following the removal of malpositioned titanium plates, the release of scarred orbital tissues, and the anatomical restoration of the orbit, the early postoperative course showed no immediate functional deterioration. At one month postoperation, a residual exohypotropia persisted, with a slightly increased deviation angle. Diplopia was no longer subjectively reported at that stage, likely reflecting adaptive sensory mechanisms. 

As residual ocular misalignment persisted after anatomical stabilization, staged ophthalmological management was undertaken to restore functional ocular alignment. The first stage focused on correction of the vertical deviation through targeted extraocular muscle surgery, resulting in a marked improvement in vertical alignment, with only a residual micro-hypotropia of approximately 4 PD. A manifest horizontal exodeviation and mild upper eyelid ptosis persisted following this intervention.

A second stage addressed the residual horizontal deviation and eyelid malposition using standard ophthalmological surgical techniques.

**Figure 5:**
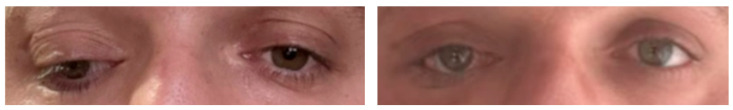
Preoperative appearance (**left**) and six months after revision orbitomaxillary reconstruction (**right**). The image at six months corresponds to one month after staged strabismus and ptosis surgery.

At six months following revision orbitomaxillary reconstruction and one month after completion of staged ophthalmological management, the patient achieved stable ocular alignment in primary gaze, with resolution of diplopia and abnormal head posture, the recovery of gross stereopsis, and symmetric eyelid position (Figure 5).

Patient-reported orbital pain decreased from 7/10 preoperatively to 0-1/10 on the VAS at final follow-up (Table 1). Overall, substantial functional improvement and restoration of facial symmetry were achieved.

### 3.3. Orbitomaxillary Stability and Implant Position

Postoperative clinical and radiological evaluations demonstrated stable implant positioning and restoration of normal orbitomaxillary anatomical relationships. Both implants remained in the planned position, without evidence of displacement, migration, or malalignment.

**Figure 6:**
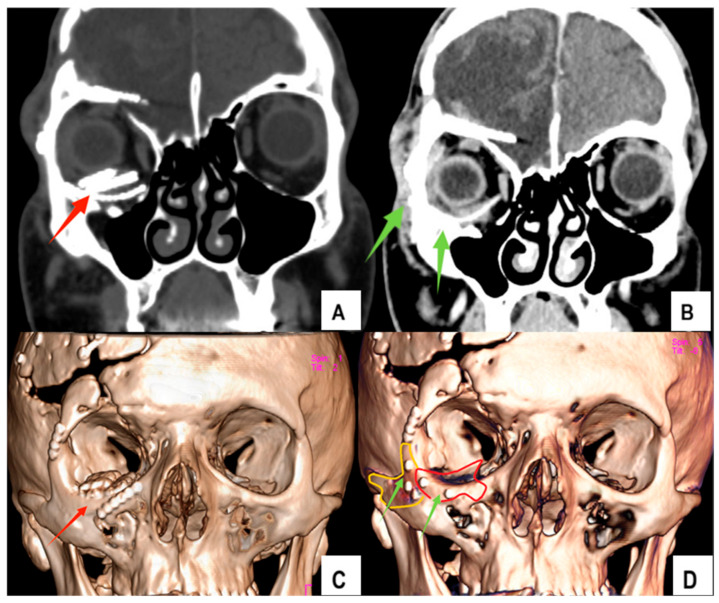
Preoperative CT and 3D reconstruction showing malpositioned multiple titanium plates (**A**,**C**); (red arrows). Postoperative CT and 3D reconstruction at 6 months demonstrating personalized PEEK and PDMS implants in the planned position (**B**,**D**); (green arrows). PEEK implant projection is highlighted in red (**D**) and PDMS projection in orange (**D**).

Follow-up computed tomography (CT) confirmed the accurate reconstruction of the orbital floor, the restoration of orbital volume, and symmetric malar projection relative to the contralateral side (Figure 6). Globe projection asymmetry decreased from 3.5 mm preoperatively to <1 mm at six months. Clinical examination and standardized photographs showed stable facial contours and improved facial symmetry, with no visible deformities or irregularities (Figure 5).

No implant-related complications, including displacement, migration, infection, or foreign body reaction, were observed during follow-up. Overall, the findings demonstrated sustained structural stability of the orbitomaxillary reconstruction, with maintained implant positioning and improved anatomical symmetry at six months.

## 4. Materials and Methods—Literature-Based Translational Rationale

This section presents a focused narrative synthesis of relevant in vitro and clinical evidence on PDMS and PEEK biomaterials and contextualizes material selection within an evidence-based translational framework.

### 4.1. Scope and Search Strategy

A focused literature review was conducted (PubMed/Medline, no language limits, and latest search August 2025) to identify in vitro and preclinical evidence relevant to the biocompatibility, cytotoxicity, and long-term stability of implant-grade PDMS in head-and-neck applications and ISO 10993-aligned assays, following established methodological principles for narrative reviews [20]. Search terms included combinations of “PDMS”, “polydimethylsiloxane”, “PEEK”, “craniofacial implants”, “biocompatibility”, and “ISO 10993”. Studies were screened for relevance to long-term implantable medical-grade materials, with prioritization of head and neck applications. Additional narrative and device-class sources (textbook/encyclopedic chapters, manufacturer datasheets) were screened for contextual properties and clinical usage patterns of PDMS and PEEK facial implants. The term “ISO 10993” was not included in the initial search string but was applied as an interpretative framework during study selection to identify studies reporting cytotoxicity and biocompatibility outcomes consistent with ISO 10993-aligned methodologies.

### 4.2. In Vitro Cytocompatibility of PDMS (ISO 10993 Aligned)

Multiple in vitro studies report an absence of cytotoxicity for PDMS-based formulations using ISO 10993-5/-12-type assays (e.g., MTT, extraction, and direct contact) across epithelial and stromal cell lines relevant to craniofacial tissues, supporting PDMS as being largely bioinert when in direct contact with living cells. Similar non-cytotoxic profiles have been reported across different PDMS applications [21,22].

### 4.3. Physicochemical and Surface Considerations

Comprehensive reviews of silicone-based biomaterials describe PDMS as chemically inert, hydrophobic, elastomeric, and thermally stable, features linked to low protein adsorption and modest baseline cell adhesion. Taken together, these properties explain its clinical record of tissue compatibility and long-term stability when used as an implant elastomer [6,23,24]. In the context of midfacial contour restoration, these characteristics are particularly relevant for achieving soft-tissue conformity without excessive stress shielding or contour rigidity.

### 4.4. Evidence from the Head-And-Neck and Facial Implant Literature

Head-and-neck overviews list silicone elastomers among the standard alloplastic options for soft-tissue contouring of the midface (malar), with complication rates driven more by pocket technique, fixation, and patient factors than by inherent cytotoxicity. Contemporary summaries in ENT and facial plastics indicate that silicone facial implants are clinically acceptable when appropriately sized and properly fixated to limit migration—considerations directly relevant to the present malar augmentation approach [25,26].

### 4.5. Complementarity with PEEK for Hybrid Reconstruction

The parallel literature on PEEK in maxillofacial reconstruction documents excellent biocompatibility, radiolucency, and mechanical stability in orbital/midface use [5,27]; however, its rigidity limits soft-tissue conformity—hence the translational rationale to pair rigid PEEK (structural support of orbital floor) with elastic PDMS (volumetric malar contour) in revision settings [28]. This “graded-stiffness” concept reflects the mechanical gradient between bone and overlying soft tissues and aligns with contemporary reconstructive biomaterials principles [29,30]. 

### 4.6. Material Specification and Regulatory Context

Manufacturer documentation for NuSil implant-line silicones and specific listings for MED-4244 identify medical-grade, implant-appropriate formulations with established healthcare use; while not substitutes for peer-reviewed data, such technical sheets support process control, sterilization compatibility, and primer/fixation guidance, complementing the existing peer-reviewed safety record [16]. 

### 4.7. Translational Takeaway for This Case

Taken together, available in vitro and device-class data indicate that implant-grade PDMS demonstrates low cytotoxic potential, a bioinert profile, and stable elastomeric properties in long-term applications. When appropriately sized, securely fixated, and placed within a well-designed surgical pocket, silicone elastomers have shown acceptable safety profiles in selected craniofacial applications [31]. In the present case, hybrid combination with PEEK leveraged mechanical complementarity between rigid orbital support and compliant malar contour restoration [27,29,32]. PEEK restored orbital structural volume with radiographic clarity, while PDMS enabled elastic, natural contour adaptation without additional osteotomies, thereby providing a literature-informed translational framework for the present revision approach.

## 5. Discussion

This report presents a complex revision orbitomaxillary reconstruction using a hybrid, patient-specific approach that combines rigid PEEK for orbital floor support with elastic PDMS for malar volumetric restoration, supplemented by autologous micro-fragmented fat grafting. 

Post-traumatic malar deformities frequently include a significant soft-tissue component that cannot be fully corrected by rigid implants alone. While PEEK restores structural support, stiffness mismatch may affect contour transitions, and autologous fat grafting—although commonly used—shows variable long-term volume retention. Therefore, a compliant material such as PDMS may provide a more stable volumetric interface, complementing skeletal reconstruction and improving soft-tissue conformity [32,33,34]. 

In contrast to orbital reconstruction, where volume overcorrection is frequently recommended, the present case involved a compliant PDMS implant intended for soft-tissue augmentation. Given the expected postoperative tissue adaptation and capsule formation around silicone-based materials, a slight undercorrection was intentionally applied to reduce the risk of overprojection. However, we acknowledge that this approach is not yet supported by robust comparative clinical evidence and should be interpreted as a case-specific strategy [6,33,34].

The rationale for this material pairing was deliberately literature-based: instead of relying on proprietary in vitro data, Section 4 synthesizes published evidence on PDMS biocompatibility, chemical stability, and elastomeric behavior alongside the well-documented PEEK clinical performance in craniofacial reconstruction [16,30]. It should be emphasized that this report does not introduce a novel silicone formulation; rather, it clinically translates an established class of implant-grade PDMS supported by ISO 10993-aligned cytocompatibility data and decades of documented in vivo medical use.

The increasing integration of three-dimensional printing and patient-specific implants in craniofacial surgery has been well documented in recent systematic reviews and clinical reports, highlighting their versality across reconstructive indications and supporting their growing clinical adoption [32,35].

Previous clinical experience with PDMS as a permanent implant material in humans supports its translational potential. In “The VaMa” study, a PDMS-based elastomeric capsule was implanted in aphakic eyes without adverse events or inflammatory reactions [6,31,36]. On the skeletal side of reconstruction, the PEEK literature consistently reports excellent biocompatibility, radiolucency, and dimensional stability in orbital and midfacial indications, while also acknowledging that rigidity can limit soft-tissue conformity—precisely the gap addressed here by adding PDMS [5,27]. 

Although PEEK is often associated with load-bearing applications, its use in orbital reconstruction is well established due to its dimensional stability, radiolucency, and ability to reliably restore orbital volume in geometrically complex but non-load-bearing anatomical regions. 

The clinical sequence in our patient matched this translational logic. The removal of five titanium plates, the decompression and disincarceration of the inferior rectus and inferior oblique muscles, and PEEK-based orbital floor reconstruction restored anatomy and volume without imaging artifacts [28]. The PDMS malar implant, designed by CT mirroring, provided elastic, non-destructive volumetric augmentation. This approach avoided the need for additional osteotomy or bone repositioning, thereby reducing surgical trauma compared to conventional zygomatic repositioning required for the correction of depressed fractures and yielding natural contour transitions while preserving existing bone [35]. From a technical standpoint, the incorporation of pre-designed screw holes during digital planning may enhance fixation reliability, particularly when fixation is required near implant margins, thereby reducing the risk of intraoperative edge chipping or material damage. Autologous micro-fragmented fat grafting was used to support soft-tissue integration and further refine contour restoration [33]. Fat grafting was used as an adjunctive technique to refine minor contour irregularities and improve soft-tissue integration, whereas the PDMS implant provided stable and predictable volumetric augmentation.

The postoperative course was anatomically uneventful with stable implant positions and no signs of infection, extrusion, or inflammatory reaction throughout the six-month postoperative period. Functionally, although passive ocular motility of the right eye improved after decompression, a residual vertical diplopia persisted due to exohypotropia caused by partial oculomotor paresis. It was successfully eliminated by secondary strabismus surgery restoring binocular single vision in primary gaze. This staged correction underlines the importance of multidisciplinary rehabilitation in orbitomaxillary trauma and does not detract from the robustness of the implant-based reconstruction.

It should be acknowledged that the use of silicone-based facial implants has historically been associated with specific clinical risks, including implant migration, capsule formation, and late infection, particularly when non-patient-specific implants are used or when fixation is inadequate. While earlier silicone-based materials such as Silastic (Dow Corning) were associated with these complications, contemporary medical-grade PDMS differs in terms of purity, manufacturing control, and clinical application context. In the present case, several factors were deliberately employed to mitigate these risks, including patient-specific implant geometry derived from CT-based mirroring, secure rigid fixation to the underlying bone using titanium screws, and placement within a well-defined surgical pocket, all of which may reduce the likelihood of implant migration and related complications. Nevertheless, the favorable outcome observed in this single revision case should not be interpreted as a basis for indiscriminate generalization. Careful patient selection, meticulous surgical technique, and long-term follow-up remain essential when considering PDMS as a permanent implant material in the cranio-maxillofacial region.

Two limitations deserve emphasis. First, follow-up was six months, which we regard as medium-term validation sufficient for a translational case report, but insufficient to define long-term performance. Extended clinical follow-up is ongoing and planned to further evaluate long-term implant stability, functional outcomes, and the potential for late complications. Second, we did not include original cytotoxicity experiments in this article; rather, Section 4 purposefully leans on published in vitro data to justify clinical safety and material choice [6,24,31].

In aggregate, this case provides evidence-informed, first-in-practice support for a graded-stiffness strategy in revision orbitomaxillary reconstruction—PEEK for durable structural support and PDMS for soft-tissue-mimetic contouring—implemented via CT-based mirroring and patient-specific design. Literature-derived in vitro safety data for PDMS, together with the established clinical record of PEEK, justify this hybrid approach and frame a credible translational bridge from biocompatible materials science to the clinical restoration of anatomy, function and aesthetics [5,16,25,27,31].

## 6. Translational Significance

This case highlights the translational potential of combining rigid PEEK with elastic PDMS in a patient-specific, graded-stiffness construct for cranio-maxillofacial reconstruction. The approach highlights a biomaterials-driven pathway from established evidence to clinical application.

## 7. Conclusions

This case illustrates the feasibility of combining patient-specific PDMS and PEEK implants in revision orbitomaxillary surgery. The hybrid construct was associated with the restoration of structural support and soft-tissue contour, with stable anatomical and functional outcomes at six months. Although longer follow-up and larger patient cohorts are required to confirm long-term durability, the present findings support a graded-stiffness biomaterial strategy as a plausible translational framework for complex craniofacial reconstruction.

## Figures and Tables

**Table 1:** Objective preoperative and postoperative parameters.

Parameter	Preoperative	6 Months Follow-Up
VAS Pain	7/10	0–1/10
Vertical Deviation	30 PD *	0–4 PD
Horizontal Deviation	30–35 PD	0 PD
Globe Projection Asymmetry	3–4 mm	<1 mm

* PD = prism diopter.

## Data Availability

The original contributions presented in this study are included in the article. Further inquiries can be directed to the corresponding authors.
